# Mobility and Participation of People With Disabilities Using Mobility Assistive Technologies: Protocol for a Mixed-Methods Study

**DOI:** 10.2196/12089

**Published:** 2019-04-16

**Authors:** François Routhier, W Ben Mortenson, Louise Demers, Atiya Mahmood, Habib Chaudhury, Kathleen A Martin Ginis, William C Miller

**Affiliations:** 1 Department of Rehabilitation Université Laval Québec, QC Canada; 2 Centre for Interdisciplinary Research in Rehabilitation and Social Integration Centre Intégré Universitaire de Santé et de Services Sociaux de la Capitale-Nationale Québec, QC Canada; 3 Department of Occupational Science and Occupational Therapy University of British Columbia Vancouver, BC Canada; 4 Rehabilitation Research Program GF Strong Rehabilitation Centre University of British Columbia Vancouver, BC Canada; 5 School of Rehabilitation Université de Montréal Montréal, QC Canada; 6 Centre de Recherche de l'Institut Universitaire de Gériatrie de Montréal Centre Intégré Universitaire de Santé et de Services Sociaux du Centre-Sud-de-l’Île-de-Montréal Montréal, QC Canada; 7 Department of Gerontology Simon Fraser University Burnaby, BC Canada; 8 School of Health and Exercise Sciences University of British Columbia Kelowna, BC Canada; 9 Department of Medicine University of British Columbia Kelowna, BC Canada; 10 Rehabilitation Research Program Vancouver Coastal Health Research Institute Vancouver, BC Canada

**Keywords:** mobility limitation, physical barriers, social participation, assistive technology

## Abstract

**Background:**

Many community-dwelling individuals living with a disability use mobility assistive technologies (MATs). MAT devices are generally beneficial for individuals with mobility impairments. However, less is known about the specific factors that may foster or deter mobility and community participation.

**Objective:**

The purpose of this protocol is to describe the methodology for a study including three main objectives: (1) to understand the places people using MAT go and the things they do, (2) to identify perceived barriers and facilitators as well as users’ desired environmental modifications, and (3) to understand subjective and objective issues related to environmental accessibility.

**Methods:**

A mixed-methods study was conducted in Vancouver and in Quebec City. Qualitative interviews were conducted to address all three objectives. In addition, Objective 1 was achieved through collection of global positioning system (GPS) data and activity diaries with 36 participants per site who represented six types of MAT users (ie, cane, walker, crutches, manual wheelchair, power wheelchair, and scooter). All participants were invited to take part in all aspects of data collection. PhotoVoice was used to address Objectives 2 and 3. Two environmental audits were used to address Objective 2. The Stakeholders’ Walkability/Wheelability Audit in Neighbourhood (SWAN) measured perceptions related to a variety of community environmental features associated with mobility and participation. A total of 24 participants were recruited to each study site for SWAN data collection. The Measure of Environmental Accessibility (MEA) was also used to objectively measure access to exterior and interior environments selected earlier in the project by the participants that could benefit from improvements.

**Results:**

Funding for this study was obtained from the Social Sciences and Humanities Research Council of Canada. Approval was obtained from the University of British Columbia Research Ethics Board and the *Centre intégré universitaire de santé et de services sociaux de la Capitale-Nationale* Research Ethics Board. Regarding the MEA evaluations, 19 locations (ie, buildings and exterior spaces) where obstacles have been identified by the participants of the PhotoVoice focus groups have been evaluated in Quebec City and 20 locations have been identified in the Vancouver region by the participants of the community forums. Data collection for this project was completed in December 2018. Analysis and writing of manuscripts are underway.

**Conclusions:**

The use of a variety of methods to gather data on participation and mobility will allow a more holistic consideration of factors influencing mobility with a MAT device. This study will provide objective information about the mobility of participants and identify barriers and facilitators that impact their mobility and community participation. Through the mixed-methods approach employed in this study, we will gain a subjective evaluation of the participants’ neighborhoods, including personally meaningful information on environmental features that influence participants' everyday mobility and participation. We will also gain an objective evaluation of particular obstacles that community users of MAT identify as significant barriers to their ability to access public environments. We anticipate that these findings will help to identify a broad spectrum of solutions to improve the mobility and community participation of MAT users.

**International Registered Report Identifier (IRRID):**

DERR1-10.2196/12089

## Introduction

In 2012, over 7% of Canadians aged 15 years or older (ie, approximately 1,971,800 individuals) reported having a mobility-related disability [1]. According to Charette et al, approximately 1,125,000 community-dwelling individuals aged 15 years or older used walking aids, representing 3.2% of the Canadian population [2]. Of these individuals, 962,290 used canes, walking sticks, or crutches and 465,340 used a walker [2]. Approximately 1% of Canadians (ie, approximately 288,800 individuals) used wheeled mobility devices (eg, scooters and manual or powered wheelchairs) for their daily activities [3]. Mobility assistive technologies (MATs) have been associated with increased mobility [4-7], defined as all movements leading to a change in position or location of an individual by his or her own means [8]. Moreover, some research has indicated that MAT use increases independence [4,5,9] and community participation [9-11].

Unfortunately, there are a variety of issues related to the use of MAT, such as accessibility to buildings via doors, accessibility to restrooms [12-14], and absence of sidewalks or of curb cuts [15-17], inducing physical strain on individuals who have to overcome these barriers. These issues reduce MATs’ potential impact on mobility, independence, and community participation. Although access to MAT is recommended by the World Health Organization to improve the lives of people with disabilities [18], many users do not receive the devices they need and fewer users receive the MAT training they require to effectively use the devices [19-21]. For example, it is estimated that half of all manual wheelchair users in Canada need assistance for propulsion [10], which likely affects their independence. Moreover, social stigma may represent a barrier for some MAT users [22-25]. In Canada and many other contries, there is a wide variety in how MAT are funded. In 2006, out of the entire Canadian adult population with mobility disabilities who used MAT, 15.2% needed more aids (ie, some needs met) and 10.5% had none of the required equipment (ie, no needs met) [26]. Furthermore, despite several legislative changes, individuals using MAT still frequently encounter accessibility problems [5,11,27-33]. Laws regarding the built environment are intended to foster social participation and equal access rights for people with disabilities, however, they do not address all potential barriers and, thus, many environmental barriers persist [34,35].

There is a general understanding of the characteristics of MAT users’ mobility (ie, distances travelled and encountered obstacles); however, we know little about how MAT influences users’ community participation, here defined as “the involvement of people in a geographic community that includes mobility, daily activities, work, and social engagement” [36]. Different methods of documenting travel habits and environments should be explored with MAT users to find a combination of measures that allows a thorough assessment of their community participation and daily activities. Although many authors have discussed the potential for objective measures using global positioning systems (GPS) and data loggers to capture real-time mobility [37-41], few studies have reported such data [39-41]. Furthermore, these data alone do not provide a complete picture of MAT [39-41], as they do not take into account the individuals’ lived experience. Although some authors have measured the frequency of participation and perceived limitations among wheelchair users [42,43], there is scarce information about their day-to-day participation.

It is therefore critical to study MAT users’ mobility in a more comprehensive and in-depth manner in relation to their community participation. By identifying barriers to MAT users’ mobility, this study aims to create a more inclusive society for all. With this overarching goal, we present a mixed-methods study with the following objectives:

1. To understand the places MAT users go and the things they do.
2. To identify perceived barriers and facilitators as well as the users’ desired environmental modifications.
3. To understand subjective and objective issues related to environmental accessibility.

## Methods

### Overview

This project used a participatory approach [44-47] that involved collaborators from municipalities and the community who were concerned with the daily lives of citizens with disabilities. The aim was to create a partnership that enabled the implementation of concrete actions and modifications that facilitated community participation among individuals with disabilities. A participatory approach was used to encourage the identification of solutions to overcome barriers to community participation, including improvements to MAT design; provision and training in MAT use; or the development of policies, regulations, actions, or services to improve the mobility of individuals with disabilities.

### Participants

To qualify for the study, participants must have lived in Metro Vancouver, New Westminster, or North Vancouver, British Columbia, or in Quebec City, Quebec, and the surrounding area. Participants were required to use a MAT as their primary means of mobility, which could have been a cane, walker, crutches, manual wheelchair, power wheelchair, or scooter. Participant demographics (ie, age, type of disability, job, gender, or technological affinity) were not considered as eligibility criteria to facilitate the recruitment procedure. Individuals were excluded from the study if they were unable to communicate in French or English or if they could not provide informed consent. People who lived in nursing homes or residential care facilities were also excluded from the study.

### Study Design

To address the above-mentioned objectives, a mixed-methods approach was proposed. All participants took part in a semistructured interview, provided demographic information, and completed the standardized measures. Participants also had the option to participate in three additional methods: (1) GPS tracking of participants’ movement in the community combined with an activity diary, (2) PhotoVoice, and (3) physical environmental audits (see Table 1 for a detailed description of the relationship between the methods and the study objectives).

### Procedure and Data Collection for Global Positioning System Tracking, Activity Diary, and Qualitative Interview

Participants’ mobility was recorded using a portable GPS (Travel Recorder XT, model BT-Q1000XT, Qstarz International Co). These data were supplemented with an Apple iPad mini (model ME280C/A, Apple Inc)-based activity diary app (ie, the customized Filemaker Go app) that allowed participants to describe the places they visited, their activities, the modes of transportation they used, and whether they were accompanied by others (see Multimedia Appendix 1 for details). The participants took part in a training session on how to use the app and the GPS. Participants were given a troubleshooting document for the devices and contact information for the research assistants in case of any difficulty with the devices and app or for emergencies. Research assistants were also available during the data collection process to answer any questions regarding the equipment and process. The participants used the GPS and app for a 1-week period. Upon completion of the data collection process, the participants returned the devices to the research assistants who reviewed the data (ie, tracks from GPS data and the diaries) with them. The research assistants then conducted a 20-minute, qualitative, semistructured interview with the participants regarding their main MAT and other MATs used.

**Table 1:** Research questions and methods.

Methods	Study objectives	Subresearch questions
GPS^a^ mobility data + activity diary + qualitative interview	1. Understand the places people go and the things they do	Where do people who use different types of MAT^b^ go? Where do people who use different types of MAT not go? What activities do they recall doing and what device were they using? When do they do these activities? Where do they do these activities?
PhotoVoice (includes qualitative interviews and focus groups)	2. Identify perceived barriers and facilitators as well as the users’ desired environmental modifications	What barriers to mobility and social participation do people who use different types of MAT encounter? What facilitators to mobility and social participation do they encounter?
	3. Understand subjective and objective issues related to environmental accessibility	What changes would they like to see happen to improve their mobility and social participation? How would they like to see these changes facilitated?
Adapted SWAN^c^ tool (subjective audit) + MEA^d^ (objective audit)	2. Identify perceived barriers and facilitators as well as the users’ desired environmental modifications	How walkable or wheelable is the selected block? What positive or negative elements are identified? How accessible is public infrastructure when visited by device users? What positive or negative elements are identified?

^a^GPS: global positioning system.

^b^MAT: mobility assistive technology.

^c^SWAN: Stakeholders’ Walkability/Wheelability Audit in Neighbourhood.

^d^MEA: Measure of Environmental Accessibility.

To describe the sample, the research assistants also gathered quantitative data, including demographic information and the following outcome measures:

1. Hospital Anxiety and Depression Scale (HADS) [48]: auto-administered measure evaluating and screening of potential anxiety and depression cases; measure is divided into two scales of seven items; rating is on a scale of 0-3; a score is generated for each subscale and for all items.
2. Self-report Late-Life Function and Disability Instrument [49]: assessment of meaningful change in function and disability; frequency and capability of performing life tasks are measured.
3. Life-Space Assessment (LSA) [50]: 20-item questionnaire measuring mobility areas (ie, home, around the home, neighborhood, city, and outside the city) while considering interactions between the person and the environment.
4. Mobility Device Use Confidence [51,52]: 65-item self-report questionnaire designed to measure confidence with mobility device.
5. Social capital measure [53]: measure examining social and behavioral determinants of health and well-being.

The data will also be combined with anxiety and depression data in predictive analyses and will be included in analyses to determine the influence of personal, MAT, and environmental factors on the activity spaces of people who use MAT using the GPS and trip diary data. Each method reported in this protocol requires separate analyses. To avoid bias, the interview guide (see Multimedia Appendix 2) was developed by team members prior to the data collection and all interviewers received training. The interview guide allowed participants to describe their own perception of barriers and facilitators to their mobility. The interview guide was developed to help participants provide their personal experience. According to participants’ addresses, a walking score was calculated using the website Walk Score [54], which is available for every address in the United States, Canada, and Australia. The tool ranks cities and neighborhoods according to the level of walkability, taking into account public transit, better commutes, and proximity to people and places. Our objective was to recruit 36 participants per site (n=72 total), representing 6 users from both sites per type of MAT (ie, cane, walker, crutches, manual wheelchair, power wheelchair, and scooter). Participants were invited to take part in other data collection methods.

### Procedure and Data Collection for PhotoVoice

PhotoVoice is a community-based participatory research method through which participants are asked to record visual images that capture their lived experiences [55,56]. It facilitates participant empowerment by considering investigators and participants as equal partners in the research process, such that participants are recognized as experts of their own experiences. Through a four-step PhotoVoice process, participants completed training, individual interviews, GPS data collection, and focus groups. 

First, each participant took part in a training session on how to operate the camera feature of the app discussed in the previous method used. The training session allowed exchanges on ethical photo etiquette, specifically the importance of using a photo and video release form when taking pictures of other individuals. During the training session, participants worked with the researchers to identify potential images that they might purposefully set out to capture. Participants received a troubleshooting document for the provided device as well as the research assistants’ contact information in case of a problem with the devices and apps or an emergency. They were also provided with a folder containing a summary of the project and photo and video release forms for obtaining consent from individuals in their pictures.

Second, over a 2-week period, which could have been concurrent with the GPS data collection method, participants were asked to take pictures or videos of the mobility- and participation-related barriers and facilitators they encountered. They were encouraged to take the tablet with them at all times during this period. It was suggested that they use the app to record notes about each image, describing their reasons for taking it. At the end of the first week, the research assistants contacted the participants to check in and ask about any problems or difficulties the participants may have been experiencing.

Third, upon completion of the above-mentioned 2-week period, participants took part in an individual PhotoVoice interview to discuss their most significant photos (ie, a personal selection of a maximum of 10 images). The interviewer looked for common themes among the pictures, and discussed suggestions for improvements and how to facilitate them (see Multimedia Appendix 3). This interview lasted approximately 20-30 minutes, depending on the number of photos selected by the participant.

Finally, PhotoVoice focus groups were held until a total of 24 participants per site were recruited for this method of data collection. However, 36 participants per site completed the PhotoVoice procedure excluding the focus group, as they could have also participated in the GPS tracking, activity diary, and qualitative interview phase or they could have been different participants. After 5-7 participants who used various types of MATs finished taking pictures, they were asked to take part in a group discussion about the photos they had taken and a PhotoVoice focus group was planned (ie, approximately three focus groups per site). Participants were not required to participate in the focus groups. Participants who completed focus groups took turns sharing the most important images they had previously selected during their individual PhotoVoice interview. Participants were asked the following questions about their images and videos: “Please describe the photo/video you have chosen.” “Why did you select this photo/video for the interview?” and “Where was the photo/video taken?” Then, the entire group was asked, “Do members of the group have questions or comments about this picture?” After the photos were shared, the group was asked the following questions:

1. “What common themes do you see among your photos/videos and which photos can be grouped in those themes?” (Group photo selection)
2. “If you wanted to see any improvements made based on the images/videos that you selected, what would those be?”
3. “How would you suggest these improvements should be made?”
4. “How could the images or videos you took be used to facilitate those improvements?”

Participants that were unable to attend the focus groups were able to access the results if they were interested. The themes identified by the groups were shared among participants to gain a sense of how the findings resonated. The PhotoVoice focus groups were audio-recorded and transcribed verbatim. Images were numbered and referenced in transcripts. Each focus group lasted 2-2.5 hours.

The transcribed interviews will be analyzed using thematic analysis [57] through an inductive approach to coding. This process requires identification of relevant text in order to categorize data into emerging themes. The visual data collected from participants will be used to supplement the thematic analysis.

At the end of the study, a photo exhibition will be held to celebrate the participants’ work and raise public awareness of relevant issues. Family, friends, relevant stakeholders, and the general public will be able to view the pictures and listen to the stories behind them. The exhibition will be held in local libraries, community centers, or other public venues. Participants interested in taking part in the photo exhibition will be asked to select photos from the previous focus groups and write captions to accompany them. If the participants prefer, captions can be written by the researchers and approved by email to ensure the captions match their interpretations. The participants’ photos and captions will be presented in full consultation with them. Participants will also be integrated in the planning process of the exhibition regarding the type of presentation and the date(s) and place at which the exhibition will take place.

### Procedure and Data Collection for SWAN and Measure of Environmental Accessibility

Two instruments were used as environmental audits. The first, a modified version of the Seniors’ Walkability Audit in Neighbourhood, renamed the Stakeholders’ Walkability/Wheelability Audit in Neighbourhood (SWAN) [58-60], was developed to collect objective data across five domains of the built environment: functionality, safety, destinations, aesthetics, and social aspects. The 98-item tool was designed to conduct microscale audits of street segments or blocks between two intersections. In addition to the checklist, participants were asked to take photographs to document barriers or facilitators to walkability and wheelability found in their neighborhood. The SWAN tool also included a secondary form to record additional contextual information that was not taken into account in the audit tool, such as general land use of the area. The original measure was pilot-tested with 24 older adults in three neighborhoods in Frankfurt, Germany, to assess its acceptability and utility and to collect pilot data on microenvironmental features (eg, sidewalk quality and street lighting) [61]. In this study, one research team member was paired with a MAT user to audit four segments in his or her neighborhood. Segments consisted of street sections between two intersections that were purposefully chosen by the participant to highlight barriers and facilitators to their daily mobility. In this way, the SWAN tool helped participants identify areas and elements of the built environment that could be improved for mobility and participation outcomes among MAT users. In our study, 24 participants per site were targeted for this method of data collection.

The SWAN data collection procedure was divided into three parts. The first part consisted of training the participants in how to use the SWAN tool. During the training sessions for the SWAN, participants received a Google Maps image of their own neighborhood. They were asked to identify four segments that they wanted to assess using the tool. The participants chose their own segments according to the following criteria: (1) segments that represented their neighborhood and had environmental features that had barriers or facilitators to maneuvering around with their MATs and/or (2) segments on streets that they frequently traveled.

The research assistants ensured that selected segments were auditable within a 2-hour period (ie, the chosen segments were not too far from one another). The four segments did not necessarily have to be in close proximity to the participant’s house nor were they required to have automobile traffic. The selected segments could be *incomplete* (ie, due to temporary maintenance work of one sidewalk or crosswalk).

The second part of data collection with the SWAN consisted of user-led data collection for each of the selected segments. Each participant was accompanied by a research assistant during data collection who helped take pictures of environmental factors, while conducting simultaneous audits of the segments. If participants wanted to change a previously selected segment on the day of data collection, they were permitted to do so as long as the new segment was not located at a distance too far from the other three.

The third and last part of data collection with the SWAN was a community forum. Community forums were held to share preliminary findings with the SWAN study participants and stakeholders. Although, it was not mandatory for each participant to attend the community forum, they were encouraged to do so. Invited stakeholders were selected from citizen committees, advocacy groups, city planners, or other community organizations working in the fields of disability, accessibility, and mobility. Members of the advisory committee who serve in the study sites were also invited. These forums fostered dialogue and discussion around methods for facilitating knowledge translation of SWAN findings and identifying potential intervention sites and strategies in each city.

The second audit tool was the Measure of Environmental Accessibility (MEA) [62], which required the research assistants to rate the indicators of the environments identified for improvement by the participants. The MEA was briefly presented to the participants during the PhotoVoice focus groups in Quebec City and during community forums in Vancouver. It included observable and measurable features of the built environment that were considered valuable indicators of accessibility to urban infrastructure for adults with disabilities. The MEA assessed exterior and interior urban built environments, including seven types of urban infrastructures: parking lots, pedestrian facilities, building access from the exterior, access to equipment, interior maneuvering areas, places for learning and leisure, and public restrooms [62]. The MEA labels were deconstructed to create three categories of information: (1) elements (ie, what was going to be evaluated), (2) components (ie, subcategories refining the description), and (3) criteria (ie, what needed to be measured) [62]. The rating scales included the following: (1) actual measures (ie, observable measures in the environment), (2) compliance (ie, regarding an observed measure with the criterion provided for each item—absent, compliant, or not compliant), and (3) observations and modifications (ie, explanations of the observations made and information on possible modifications to be made to improve accessibility) [62]. Most items had good-to-excellent interrater reliability indicators (626/882, 71.0%, using Gwet’s agreement coefficient) [62]. Participants who took part in the PhotoVoice focus groups in Quebec City and the community forums in Vancouver were asked whether certain pictures presented environments to be evaluated with this measure or if other environments that were not included in the pictures should be evaluated. The gathered data from the MEA evaluations will be analyzed by identifying the most recurrent obstacles and by comparing those found in Quebec City and in Vancouver.

### Ethics

The protocol for this study was approved by the Research Ethics Boards at the University of British Columbia (approval number H15-01340), the *Centre intégré universitaire de santé et de services sociaux de la Capitale-Nationale* (approval number 2015-424), and the regional health authorities of each site. All study participants provided informed consent.

## Results

Funding for this study was obtained from the Social Sciences and Humanities Research Council of Canada. A summary of recruitment and completion of the different steps of the project can be found in Table 2. As for the MEA evaluations, 19 locations (ie, buildings and exterior spaces) where obstacles have been identified by the participants of the PhotoVoice focus groups have been evaluated in Quebec City and 20 locations have been identified in the Vancouver region by the participants of the community forums. Data collection for this project was completed in December 2018. Analysis and writing of manuscripts are underway.

**Table 2:** Recruitment and completion status of the study.

Study site	Participants recruited (N)	Participants, n (%)			
		Completed GPS^a^ tracking, activity diary, and qualitative interview	Completed PhotoVoice	Completed SWAN^b^	Withdrew
Metro Vancouver	63	35 (56)	32 (51)	24 (38)	3 (5)
Quebec	41	39 (95)	40 (98)	25 (61)	1 (2)
Total (both sites)	104	74 (71.2)	72 (69.2)	49 (47.1)	4 (3.8)

^a^GPS: global positioning system.

^b^SWAN: Stakeholders’ Walkability/Wheelability Audit in Neighbourhood.

## Discussion

The purpose of this protocol is to describe the methodology for a study that includes three main objectives: (1) to understand the places people go and the things they do, (2) to identify perceived barriers and facilitators as well as the users’ desired environmental modifications, and (3) to understand subjective and objective issues related to environmental accessibility. Thus, this study should allow us to discover information regarding the following elements:

1. Describe how environmental factors influence the mobility and participation of people with disabilities using a variety of MATs.
2. Identify environmental and personal factors that influence mobility and participation among adults with mobility impairments.
3. Identify the changes these people would like implemented to improve their mobility and participation.

The use of a variety of methods to gather data on participation and mobility allows for a more holistic consideration of the factors influencing these outcomes. The GPS tracking, activity diaries, and qualitative interviews provide objective information on the whereabouts of the participants as well as their subjective reports about the activities they are participating in and the means of mobility and transportation they are using.

Second, the identification of barriers and facilitators to mobility and participation through PhotoVoice focuses on the participants’ preoccupations. An objective evaluation of the encountered obstacles judged as priorities of improvement when accessing public environments are performed from the results obtained though the PhotoVoice focus groups in Quebec City and the community forums in Vancouver via the MEA [62]. This will provide input into the most recurring obstacles in an objective and measurable fashion (ie, proposing a design ideal) and, thus, on the practical targets that could be proposed to improve access. To support the analysis and interpretation of the data collected with the SWAN tool, a scoring system was developed. Two different types of scores were produced from each evaluation of a segment: a participant score and a total score. The participant score was based on the subjective evaluation of each domain using a 5-point Likert scale. The total score was based on objective data noting the absence or presence of the barriers and facilitators in each segment. The presence of features supporting mobility obtained the highest scores, and segments with absent or mobility-impeding features obtaining the lowest scores in the total score category. Using this combination of objective and subjective evaluation of the participants’ neighborhood via the SWAN, the MAT users provided meaningful information on what is truly important to them within the built environment that influences their everyday mobility and participation.

Finally, the photo exposition and the community forum will allow participants to be heard by referring to the collected data and the sharing of their daily reality with stakeholders, the general public, and other researchers to heighten awareness on the barriers and facilitators they commonly encounter. The participants will also be encouraged to contribute to the discussions to find solutions to the most common problems.

Foreseeable limitations of this study include challenges in recruiting users with different MAT devices. During the recruitment process, two MAT devices had to be integrated into the same group since too few participants using these MAT devices participated (ie, canes and crutches). More participants had to be recruited in Vancouver to attain the same number of overall participants as in Quebec City. This is due to the fact that fewer participants in Vancouver participated in more than one method (11/63, 17%, participants completed all three methods of data collection in Vancouver versus 25/41, 61%, in Quebec City). Also, the sample of convenience may influence generalizability of the findings. The cross-sectional nature of the data only allows the consideration of one moment in time, whereas mobility likely fluctuates due to a variety of factors.

## Appendix

Multimedia Appendix 1 Activity monitoring questions.

Multimedia Appendix 2 Interview guide for all participants.

Multimedia Appendix 3 PhotoVoice interview guide.

Multimedia Appendix 4 Peer-reviewer report from SSHRC.

## Abbreviations

GPS: global positioning system
HADS: Hospital Anxiety and Depression Scale
LSA: Life-Space Assessment
MAT: mobility assistive technology
MEA: Measure of Environmental Accessibility
SWAN: Stakeholders’ Walkability/Wheelability Audit in Neighbourhood
