# Supplementary material for: Mobility and Participation of People With Disabilities Using Mobility Assistive Technologies: Protocol for a Mixed-Methods Study
Source: JMIR Res Protoc. 2019 Apr 16;8(4):e12089. doi: 10.2196/12089 (PMC6488954; doi:10.2196/12089)
Supplement: Multimedia Appendix 1 [file resprot_v8i4e12089_app1.pdf]

Multimedia Appendix 1

Activity Monitoring Questions

1) What Mobility device did you use for this trip?

2) How did you get to your destination?

- ☐ Walk/Wheel
- ☐ Car driver
- ☐ Car passenger
- ☐ Transit
- ☐ Taxi

3) Who did you travel with?

4) How did you get around once you arrived at your destination?

5) What was your destination?

6) Purpose of your trip

- ☐ Go to market
- ☐ Recreation/exercise
- ☐ Shopping (not food)
- ☐ Meet friends (social)
- ☐ Eat/drink
- ☐ Get/return book
- ☐ Walk pet
- ☐ Work/volunteer

7) Was this activity outside or inside?
