# Supplementary material for: Mobility and Participation of People With Disabilities Using Mobility Assistive Technologies: Protocol for a Mixed-Methods Study
Source: JMIR Res Protoc. 2019 Apr 16;8(4):e12089. doi: 10.2196/12089 (PMC6488954; doi:10.2196/12089)
Supplement: Multimedia Appendix 2 [file resprot_v8i4e12089_app2.pdf]

Multimedia Appendix 2

Interview Guide

1) How do you use your mobility device on a daily basis?[1]

Prompts:

a. On a typical day, what do you do with your mobility device?
b. What kinds of activities do you do with your mobility device?
c. How much time do you spend in your mobility device?
d. How has your use of the mobility device changed over time, if at all (i.e. frequency)?
e. What is the terrain like around your home?

2) What do you like most/least about your mobility device?

Prompts:

a. Does you mobility device have any specific features?
b. If so, why did you choose these features?
c. How satisfied are you with your mobility device and its features?
d. How, if at all, do you think your technology has helped you adapt to your disability?
e. How does your mobility device fit with your sense of self? (in terms of body image/ being consciously aware of the chair)
f. What is it like to drive your mobility device? (kinaesthetics, confidence, what does it feel like)
g. How, if at all, did your life change after you began to use a mobility device?

(For those getting a new device)

h. How do you imagine your life will change after you get your mobility device?

3) What difficulties do you encounter when using your device?

Prompts:

a. What barriers do you encounter when trying to get around? (outside versus inside)
b. What assistance do you need with setup/transfers and travelling with your device?
c. What are the typical problems that you experience with your device?
d. Given that you have a physical impairment that limits your ability to walk, how does your mobility device affect your ability to participate in activities? What kinds of activities do you not participate in because of your device?
e. What kinds of things have you tried to overcome these barriers?
f. Accidents?
g. What concerns do you have about becoming deconditioned with your mobility device?

4) What do you wish your mobility device could do that it currently can’t?

Prompts:

---

[1] For those not currently using a wheelchair we will ask them to envision how they will use their wheelchair and difficulties they might encounter.

a. What would you change about your current technology to improve your mobility?
b. If you could create any new technology for your device, what would it be and why?

5) What was it like getting your first mobility device?

Prompts:

a. What was your first device like?
b. How many devices have you had?
c. How long have you used mobility device?
d. What effect did it have on your life?
e. What training did you receive?

6) How has your use of mobility devices changed over time?

Prompts:

a. How has the technology of your primary mobility device changed?
b. How have you changed?
c. How has your confidence using a mobility device changed over time?
d. What changes in activities have you made since starting to use a mobility device?
e. How has your confidence using your mobility device changed over time?
