# Supplementary material for: Mobility and Participation of People With Disabilities Using Mobility Assistive Technologies: Protocol for a Mixed-Methods Study
Source: JMIR Res Protoc. 2019 Apr 16;8(4):e12089. doi: 10.2196/12089 (PMC6488954; doi:10.2196/12089)
Supplement: Multimedia Appendix 3 [file resprot_v8i4e12089_app3.pdf]

Multimedia Appendix 3

## PhotoVoice Interview Guide

The interview script below is for one-on-one interviews or small group discussions. Statements that will solely be used in the small group discussion are in brackets.

*Preamble:* Thank you for coming in today and taking the time to participate in our study. Before we begin the interview, we would like to remind you that the discussion will be voice recorded. We will let you know when the voice recording begins and ends. The interview will be guided with interview questions and you may choose to skip questions, which you do not wish to answer. Your confidentiality is important to us and we will ensure to remove any personal identifiers from the information we receive today. We also want to disclose that although personal identifiers will be removed from the study, there is a risk that you may be identifiable by the things you say.

[During this group discussion, we will explore each participant's photos or videos individually. At the end of the discussion, we will open the floor to the group and you will have the opportunity to comment on each other's photos or videos. Anything said between any two or more group members at any time is confidential and should not be discussed outside the group without permission.]

Do you have any questions before we begin?

1. Please describe the photo/video you've chosen. (repeat questions for all pictures)
    a. Why did you select this photo/video?
    b. Where was the photo/video taken?
    c. How does this photo/video relate to the barriers and facilitators you experience with mobility?

2. Do you see any common themes amongst your photos/videos?

3. Is there anything more about your photos/videos that you would like to share?

4. Would you like to display any of these images or videos as part of photo exhibit?

5. How would you like to do this? (providing captions, deciding on layout, being present during the exhibit)

*Conclusion*: Thank you for sharing your photos, videos, and stories with us today. We greatly appreciate you participating in the discussion and contributing to our study. Anything that you have shared with us today is confidential and we will make sure to remove any personal identifiers in our analysis. If you have any questions or concerns, feel free to email us at demandprojectbc@gmail.com
