# Supplementary material for: Mobility and Participation of People With Disabilities Using Mobility Assistive Technologies: Protocol for a Mixed-Methods Study
Source: JMIR Res Protoc. 2019 Apr 16;8(4):e12089. doi: 10.2196/12089 (PMC6488954; doi:10.2196/12089)
Supplement: Multimedia Appendix 4 [file resprot_v8i4e12089_app4.pdf]

SSHRC CRSH

350 Albert Street, P.O. Box 1610
Ottawa ON K1P 6G4
www.sshrc.ca

350, rue Albert, C.P. 1610
Ottawa ON K1P 6G4
www.crsh.ca

Expert Panel Report

Applicant: McMaster University
Project Director: Kathleen A. Martin Ginis
Application Number: 895-2013-1021
Project Title: Enhancing community participation in Canadians with physical disabilities: development, implementation and evaluation of a partnered strategy

Expert Panel evaluations are intended to provide the Selection committee with an in-depth analysis of the strengths and weaknesses of a proposal, so that it can determine the relative merit of a project compared to the cohort and make funding recommendations to SSHRC.

Expert Panel Agreement

- The information provided in the applications is protected by Canada's *Privacy Act* and was made available to Expert Panel members for review purposes only. The Expert Panel has therefore agreed to treat all the material from the above-mentioned files as strictly confidential. They have agreed to ensure the destruction of the said materials once their duties have been completed.
- The Expert Panel members have attested that they are not in any conflict of interest with the project directors, or any co-applicants, collaborators and/or partner institutions.
- The Expert Panel members have agreed, that the information presented in the following pages reflects the consensus reached in their deliberations on **January 31, 2014.**

The following Expert Panel members have consented to the above terms

Dr. Nancy E. Hansen, Associate Professor and Director of Disability Studies at the University of Manitoba, Canada

Dr. LaWanda H. Cook, Extension Associate/Training Specialist, Employment & Disability Institute, Cornell University, United States

Dr. Michael G. Wade, Professor, School of Kinesiology, University of Minnesota, United States

Challenge—The aim and importance of the endeavour

Strengths

- The proposal is expansive and seeks a somewhat optimistic set of both evidence based outcomes and associated changes in practice and service delivery.
- The expert panel judged that while the actual content is not original, the expectation of the "synergy" is. It also found the scope of the project is somewhat original.
- The use of the World Health Organization's International Classification of Functioning, Disability and Health (ICF), Self-Determination Theory (SDT), and Social Cognitive Theory (SCT) are appropriate and very comprehensive means for meeting the goals of the proposed project as they consider personal and environmental factors.
- The expert panel judged that the mixed methods approach should allow researchers to assess additional facilitators/barriers which may not be readily identified by the theoretical models.
- The expert panel also considered the project to have a diverse knowledge base and noted there was the possibility of cross–discipline knowledge transfer.
- The expert panel considered the proposed scope of student and emerging academic training impressive. It also noted that there were good opportunities for post-doctoral work. The expert panel noted that it appears all partners will be engaged in meaningful decision making, product development, and information dissemination and that the project has the potential for broad impact based on its partnership structure.
- The expert panel noted that the planned regular communication between partners, and measureable outcomes suggest project sustainability. It further noted that if maintained, the desired progress should be made throughout the project. Moreover, the expert panel considered the provision of a lead team focusing on each of the three domains (including academics and community partners) to be a strength of the proposal.
- While the impact on the social-science community is hard to predict, the expert panel considered the innovation within the project to be its attempt to combine both research and practice, or undertake what appears to be "participatory action research".

Weaknesses

- The expert panel considered the proposal to have basic recognition of the degree of marginalization experienced by disabled people. The approach seems a bit dated and it lacks depth. It does not appear that the team has assessed the broader environmental barriers experienced by disabled persons beyond the physical challenges.
- The expert panel found the project to be too broad and lacking specificity. It also expressed concern that the team was spreading itself too thin over too many elements. Finally, it questioned how the team will link all the diverse aspects together in the end of the project.
- It was not clear to the expert panel how the proposal would go beyond what has already been done in this field. It noted that the bulk of this type of research dates back to the late 1980s and early 1990's in disciplines such as psychology, sociology and social work. The proposed research does not introduce new concepts to these areas, and the substantive participation of disabled persons is unclear. Moreover, the proposal lacks consideration of gender, culture poverty issues.

Challenge—The aim and importance of the endeavour

- While the significance of proposed research is well-substantiated, it is not very contemporary based on the literature review and the project director's prior work. Its range covers standard textbook references, published reports and empirical articles. Moreover, the expert panel noted that the literature pertaining to disability studies was largely absent.
- The expert panel judged the theoretical approach to be poorly conceived. It noted that the theoretical approaches chosen seem to focus on Deci and Bandura, which are not especially new. It also noted that empirical efforts with respect to social-psychology are limited by the nature of the data collected; and surveys and questionnaires do not always reliably predict future behavior.
- While the comprehensive theoretical framework and approaches should enable significant information gathering related to the three domains (employment, mobility and sport), the expert panel found that it was not as clear how they would be specifically applied to the area of employment participation.
- The expert panel noted that the project needs more realistic and/or the inclusion of increased "pilot data" to support the proposed "synergy" between empirical results and how that can be directly applied to both policy and service delivery. Moreover, if such data are available it does not appear in the proposal. While the project director notes the difficulty of producing the data referenced in notes #102 and #103, on account of small sample sizes and low power, the expert panel questioned whether a "promissory note" to increase sample size and power could produce more reliable "pilot" data? As a result of the lack of sufficient base data the expert panel also noted that the proposal provides no guarantee of a successful outcome given the time span.
- There was mention of great interest in the development of products following prior related research by the project director with persons with spinal cord injuries. The expert panel questioned how many such requests have been made and by which organizations. They also questioned to what extent these requests represent other types of disabilities, noting that this information would further strengthen the case for conducting this research and for projecting its potential impact.

Feasibility—The plan to achieve excellence

Strengths

- The expert panel found that the staggered timelines for the different thematic areas and the fact that each area has its own team should allow the partnership to meet its stated objectives in a timely manner. It also noted that the visual representation of how and when different aspects of the project would be completed was helpful.
- The expert panel noted that the prior related work of the project director and several of the partners is a strength given their successful and fruitful working relationships. Moreover, the key partners appear committed having already engaged in important pre-work for their project participation.
- Providing that the project director can keep everyone in collaboration, the expert panel noted that the wide group of scholars, active researchers, appropriate agencies, and in-kind support from participating institutions and universities speaks both to the hard work and vision of what a long-term project of this kind *might achieve.*

Feasibility—The plan to achieve excellence

- The expert panel noted that the process for handling differences of opinion seems to be particularly well thought out.
- The expert panel considered the explanations of budget items to be reasonable and noted that they indicate opportunities for both academic and nonacademic partners to travel in order to participate and share the results of this work.
- The expert panel also judged the financial and in-kind support to be considerable.
- The expert panel noted that there was a possibility of collaborative policy development.

Weaknesses

- The expert panel noted that application suggests that the input of partners outside of the research community will be involved at every phase of the project and that they will play a critical role in broadly disseminating knowledge gained from this project. However, there is some question as to how fully persons with disabilities will be meaningfully engaged.
- The expert panel questioned to what degree the team had considered plans for potential partner turnover. This issue was identified within the proposal as a challenge in prior research involving multiple partners, however the proposed strategies for addressing the situation (e.g., ensuring adequate communication) do not seem to be sufficient. The expert panel noted that there should be something more concrete; for example, each partner could be asked to provide a contingency plan for ensuring continued involvement in the event of staff turnover.
- On account of the broad nature of the project the expert panel noted that there was a risk of the team trying to over extend its reach. As a result, it questioned whether the core leadership had adequate skills and abilities to ensure the governance of the project.
- Given that the specific knowledge mobilization products will be determined based on findings in earlier phases of the project, the expert panel noted that it would have been helpful if the team had provided a better sense of what some of the products might look like based on what is currently known about the thematic areas and from the project director's prior work with the Spinal Cord Injury community and communication with partners.
- The expert panel questioned to what degree the "Other Contributors" would be a part of the project. It would have liked further precision as to whether there are already agreements in place, verbally or otherwise – and information about the specific ways each will be involved in the project. It also questioned if they have supported research in the past as the chart and explanation contained within the proposal are not clear.
- The nature of the partnership and involvement of the various groups of disabled people has to be clarified as there was not sufficient evidence of meaningful knowledge transfer within the proposal. As a result, the expert panel was not convinced that the disability community will have a true partnership. It noted that it would have been more reassuring if the proposal had indicated how many of the researchers and key partners have disabilities and/or could adequately assist in relevant knowledge transfer to the disability community.

Capability—The expertise to succeed

Capability—The expertise to succeed

Strengths

- The skill level and multiple areas of expertise of partners are assets to the application and the potential success of the project. Additionally, several partners have roles within their professional areas allowing access to individuals with disabilities and to others whom the project seeks to reach.
- The expert panel noted the project director has an impressive record of achievements and is well recognized academically; moreover she has a good track record in research and outreach in the broad area of this field of research and associated service delivery.
- The project director and her prior partners have demonstrated the ability to create innovative products based on research findings and to disseminate their findings within the research community. Given the number of community-based partners and previous development of products to benefit the spinal cord injury community, it seems that there are strong possibilities for contributing both within and outside of academia.

Weaknesses

- If the project partners have the skills to maintain the leadership the expert panel noted that they should be good to go ahead.

Additional Comments

This proposal is clearly an impressive, wide reaching effort to address an important public policy need. It combines both research and practice in ways that seem to get at an issue that receives a lot of "lip service" but lacks any serious effort to move laboratory research into the domain of face to face practice and ultimately to influence policy in positive ways.

The expert panel appreciated the focus on the three domain areas, and recognized their significance to the disability community.

The expert panel noted that it would have been helpful to have a clearer indication of meaningful involvement from the disability community, and that this would have strengthened this proposal.

The expert panel was not clear to what degree the team has considered the challenges posed by power inequalities between the academic partners and the disability community. The panel noted that as long as the academics provide equal voice to the partners outside of academia and the members of the disability community, the proposed project and team have the potential to contribute new and important knowledge that will significantly benefit individuals with disabilities.
